# Preparation and Characterization of Microencapsulated Phase Change Materials with Enhanced Thermal Performance for Cold Storage

**DOI:** 10.3390/ma18092074

**Published:** 2025-04-30

**Authors:** Yang Wang, Yunchuan Xu, Haojie Zhao, Ruilin Cao, Bei Huang, Lingling Xu

**Affiliations:** College of Materials Science and Engineering, Nanjing Tech University, Nanjing 211816, China; 202261103012@njtech.edu.cn (Y.W.); xycdudu@163.com (Y.X.); zhaohaojie@njtech.edu.cn (H.Z.); xulingling1964@163.com (L.X.)

**Keywords:** 1-decanol, microcapsule, emulsification, binary eutectic, thermal properties

## Abstract

Microencapsulated phase-change materials (MPCMs) with excellent thermal properties for low-temperature cold storage were developed in this study. Using 1-decanol as the core and methyl methacrylate as the shell precursor, the effects of emulsifier type and ultrasonic emulsification conditions were investigated. Styrene-maleic anhydride copolymer served effectively as a protective colloid emulsifier, producing MPCMs with high enthalpy and a well-defined, uniform microstructure. Under optimal conditions of 5 wt% emulsifier content relative to the oil phase, an ultrasonic power of 375 W, and an emulsification time of 12 min, the MPCMs exhibited a phase-change enthalpy of 126.7 kJ/kg. To further improve the thermal properties, a binary eutectic mixture was prepared by combining 1-decanol and 1-tetradecane at an optimal molar ratio (51.1:48.9). This binary-core MPCM showed a higher storage enthalpy (144.3 kJ/kg), with an increase of 13.9% compared to the single-core material (1-decanol). It also exhibited improved microstructural uniformity due to the stabilizing role of 1-tetradecane. These optimized MPCMs demonstrate phase-transition temperatures particularly suitable for low-temperature thermal storage, providing a practical and innovative technical solution for cold-chain logistics and vaccine refrigeration applications.

## 1. Introduction

The quality and safety of food and pharmaceuticals have gained increasing attention, with factors such as humidity and temperature significantly affecting their quality [1,2]. Consequently, large-scale cold-chain logistics are often required for the transportation of food and pharmaceuticals. However, cold-chain logistics typically rely on electric or mechanical refrigeration technologies, which result in significant energy consumption [3]. Particularly in developing countries, the shortage of cold storage facilities and long waiting times during the storage, transportation, and distribution of fresh produce often lead to chain breakage. This causes significant food waste and economic losses [4]. Moreover, the construction of new refrigeration infrastructure would greatly increase greenhouse gas emissions from the food cold chain. This could intensify climate change challenges and accelerate the consumption of non-renewable energy [5]. Therefore, the development of energy-efficient and environmentally friendly refrigerated transportation equipment is crucial [6].

Phase-change materials (PCMs) can absorb or release a large amount of energy within a short period during the phase-transition process [7]. Using PCMs to regulate the surrounding temperature effectively prevents the deterioration of food and pharmaceutical quality [1]. Thermal energy storage (TES) technology shows great potential for applications in cold storage [8,9]. However, most PCMs face challenges such as corrosion, volume expansion, and leakage during use [10,11]. To overcome these issues, the development of microencapsulated phase-change materials (MPCMs) through microencapsulation technology has become a widely adopted solution [12,13]. MPCMs are expected to have significant application prospects in short-distance cold-chain transportation [14], cold storage packaging [15], and air conditioning systems [16].

Most existing studies have used styrene (St), methyl methacrylate (MMA), and similar compounds as precursors for organic shells [17]. These materials exhibit excellent mechanical strength and corrosion resistance after polymerization, effectively protecting PCMs from the external environment [18]. The preparation of oil-in-water (O/W) emulsions is fundamental to microencapsulation processes such as suspension polymerization [19], in situ polymerization [20], and interfacial polymerization [21]. Thus, the emulsification step is critical to the performance of the resulting MPCMs. Sodium dodecyl sulfate (SDS) [22], octylphenol polyoxyethylene ether (OP10) [23], and styrene maleic anhydride copolymer (SMA) [24] have been widely used in the synthesis and preparation of MPCMs. However, existing research lacks a comparative analysis of the mechanisms and effects of different emulsifiers. The current emulsification method primarily relies on high-speed mechanical stirring. This approach often generates excessive foam, which negatively affects emulsification efficiency and product quality and significantly reduces microcapsule yield, while also increasing the molding failure rate [25]. Ultrasonic dispersion is an alternative emulsification method, where its effectiveness depends on parameters such as ultrasonic power and duration [26]. Despite its potential, the systematic optimization of ultrasonic dispersion as an emulsification process for preparing MPCMs has rarely been explored, leaving a critical knowledge gap [27,28].

Currently, the cold-chain transportation of food products and the storage temperature of cold-chain packages are typically maintained within the range of 2–8 °C [29,30]. This temperature range is also consistent with the cold storage requirements of 2–8 °C for pharmaceuticals, as specified in the European Pharmacopoeia (EP11) and the U.S. Pharmacopoeia (USP-NF). Research on MPCMs with phase-transition temperatures near room temperature and above has been extensively conducted [31]. However, studies on low-temperature PCMs suited for cold-chain transportation are relatively limited, with most existing research, both domestically and internationally, focusing on single organic PCMs [32]. Common low-temperature PCMs, such as 1-decanol, 1-tetradecane, and methyl laurate, have phase-transition temperature ranges that fail to fully meet the low-temperature requirements of 2–8 °C [33]. Additionally, the peak temperatures of their phase transitions often fall outside this range [34,35]. The high cost of 1-tetradecane and methyl laurate further restricts their application in cold-chain transportation. By employing the binary eutectic method, existing PCMs can be compounded to develop binary eutectic PCMs with phase-transition temperature ranges more suitable for low-temperature refrigeration [36,37]. In addition, enhancing the phase-transition enthalpy of PCMs is also a research hotspot [38]. The phase-transition enthalpy value can be effectively improved by using the binary eutectic method to optimize single phase-change materials [39]. This approach not only optimizes the phase-transition temperature range but also reduces material costs, offering a more cost-effective and practical solution for low-temperature cold-chain transportation [40].

In this study, inexpensive 1-decanol was utilized as the core material for MPCMs. The effects of emulsifier type and dosage on the performance of MPCMs were first investigated to identify the optimal emulsifier for the system. The ultrasonic dispersion emulsification process was also studied. Finally, a phase-change material with a more suitable phase-change temperature for low-temperature refrigeration storage was prepared using the binary eutectic method. The optimal emulsification process identified was then employed to prepare refrigeration storage MPCMs with an excellent thermal performance.

## 2. Materials and Methods

### 2.1. Materials

1-Decanol (98%), 1-Undecanol (98%), 1-Dodecanol (>98%), 1-Tetradecane (99%), 1-Hexadecane (99%), capric acid (99%), lauric acid (98%), 2-methylpropionitrile (AIBN, 98%), pentaerythritol triacrylate (PETRA, 96%), OP10, SDS, Span80, Tween80, and deionized water were obtained from McLean Biochemicals Co., Ltd., Shanghai, China. Methyl methacrylate (MMA, 99.5%) was purchased from Shanghai Lingfeng Chemical Reagent Co., Ltd., Shanghai, China. Styrene-maleic anhydride copolymer (SMA) was provided by Shanghai Leather Chemical Factory, Shanghai, China.

### 2.2. Synthesis Methods

O/W emulsions investigated in this study exhibited weak alkaline properties, necessitating the use of anionic or nonionic emulsifiers. The hydrophilic–lipophilic balance (HLB) was employed to evaluate the balance between the hydrophilic and lipophilic components of the emulsifiers. Optimal emulsification performance is achieved when the HLB value of the emulsifier closely matches that of the substance being emulsified [41]. For the O/W emulsions prepared in this study, suitable HLB values ranged from 8 to 18 [42]. In addition to the anionic emulsifier SMA and the nonionic emulsifier OP10, Span80, Tween80, and SDS were combined to formulate two compounded emulsifiers. The final emulsifiers used are listed in Table 1, and the formula for calculating the HLB values of the compounded emulsifiers is provided in Equation (1) [43].

(1) $$HLB=\sum \left(\omega_{i}{HLB}_{i}\right)$$

where $\omega_{i}$ is the mass fraction occupied by component i, and ${HLB}_{i}$ is the HLB value of component i.

**Figure 1:**
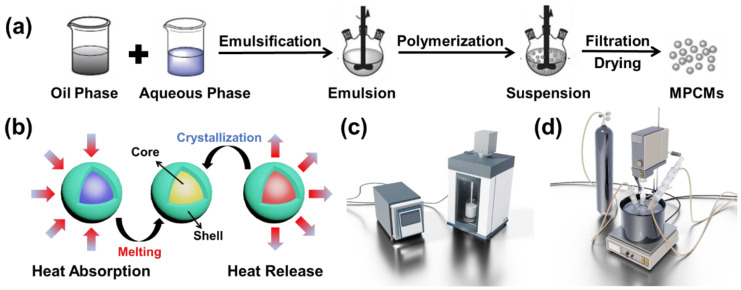
Preparation process flow diagram and equipment diagrams: (**a**) suspension polymerization process flow diagram, (**b**) MPCM working schematic, (**c**) ultrasonic emulsification device, (**d**) stirring polymerization device.

MPCMs were synthesized by suspension polymerization. The schematic diagram of the synthesis process is shown in Figure 1a. The schematic illustration of MPCMs’ operation is presented in Figure 1b. First, 0.4 g of initiator (AIBN), 5 g of shell material (MMA), 2 g of crosslinker (PETRA), and 15 g of core material were mixed as the oil phase. Subsequently, a certain amount of emulsifier was added to 150 g deionized water and dispersed homogeneously to obtain the aqueous phase, followed by mixing with the oil phase for emulsification. Emulsification was performed by ultrasonic dispersion. The ultrasonic device was operated alternately for 2 s with 2 s intervals. The experimental setup is shown in Figure 1c.

During the polymerization stage, the emulsion was transferred to a 250 mL three-necked flask. The flask was connected to a nitrogen inlet and a condensation tube at both ends, respectively, and placed in an oil bath at 85 °C; the device is shown in Figure 1d. The stirrer speed was set to 400 rpm, and the polymerization reaction was carried out for 3 h. Upon completion, the suspension was filtered to obtain the product, which was washed several times with deionized water and ethanol. The product was then dried in a blast air oven at 40 °C for 24 h.

According to Fourier’s law of heat conduction and Equation (2), heat loss per unit time decreases as the PCM phase-transition temperature approaches the ambient temperature. For a given latent heat, a phase-change range closer to 2–8 °C results in a longer cold storage duration [44].

(2) $$c\rho \frac{\partial u}{\partial t}=k\left(\frac{\partial^{2}u}{\partial x^{2}}+\frac{\partial^{2}u}{\partial y^{2}}+\frac{\partial^{2}u}{\partial z^{2}}\right)+F$$

where c is the specific heat capacity of the PCM, ρ is the density of the PCM, u is the temperature at a given position within the PCM, and F is the internal heat source of the material. The core materials of MPCMs were modified using the binary eutectic method to better meet the requirements of cold storage.

The advantage of binary eutectic PCM is that the phase-transition temperature can be adjusted by varying the mass ratio of each component. The theoretical phase-transition temperatures of the composite PCM cores were calculated and designed using the Schrader function, as shown in Equations (3) and (4) [36].

(3) $$T_{0}=\frac{T_{A}H_{A}}{{\Delta H}_{A}-T_{A}RInX_{A}}$$

(4) $$T_{0}=\frac{T_{B}H_{B}}{{\Delta H}_{B}-T_{B}RInX_{B}}$$

where T_0_ is the onset phase-transition temperature of the eutectic mixture (K), T_A_ and T_B_ denote the onset phase-transition temperatures of components A and B (K), X_A_ and X_B_ represent the mole fractions of components A and B, and R is the universal gas constant (8.314 kJ/(kmol·K)).

### 2.3. Characterization

The enthalpy of phase change was tested using a differential scanning calorimeter (DSC, Discovery DSC2500, TA Instruments, New Castle, DE, USA). The analysis began at an equilibrium temperature of −50 °C, and the temperature was increased to 50 °C at a rate of 5 °C/min. The encapsulation ratio (E) of the MPCMs was calculated based on the phase-transition enthalpy using the formula shown in Equation (5) [25]:

(5) $$E=\frac{{∆H}_{MPCM}}{{∆H}_{PCM}}\times 100\%$$

where ${∆H}_{MPCM}$ is the enthalpy of phase transition of MPCMs (kJ/kg), and ${∆H}_{PCM}$ is the enthalpy of phase transition of coated PCMs (kJ/kg).

The thermal stability of the MPCMs was analyzed using a thermogravimetric analyzer (TGA209F1, Netzsch, Selb, Germany) under a nitrogen atmosphere from 50 °C to 600 °C at a heating rate of 5 °C/min. The surface morphology of the MPCMs was observed with a scanning electron microscope (SEM, Gemini 300, ZEISS, Oberkochen, Germany). The chemical structure of the MPCMs was characterized by Fourier transform infrared spectroscopy (FTIR, Spectrum Two, PerkinElmer, Waltham, MA, USA) over the range of 4000–400 cm⁻^1^ with a resolution of 4 cm⁻^1^. A laser particle size analyzer (LPSA, Mastersizer 2000, Malvern Panalytical, Malvern, UK) was employed to determine the particle size distribution of the MPCMs.

## 3. Results and Discussion

### 3.1. Effects of Emulsification Process on Thermal Properties

**Figure 2:**
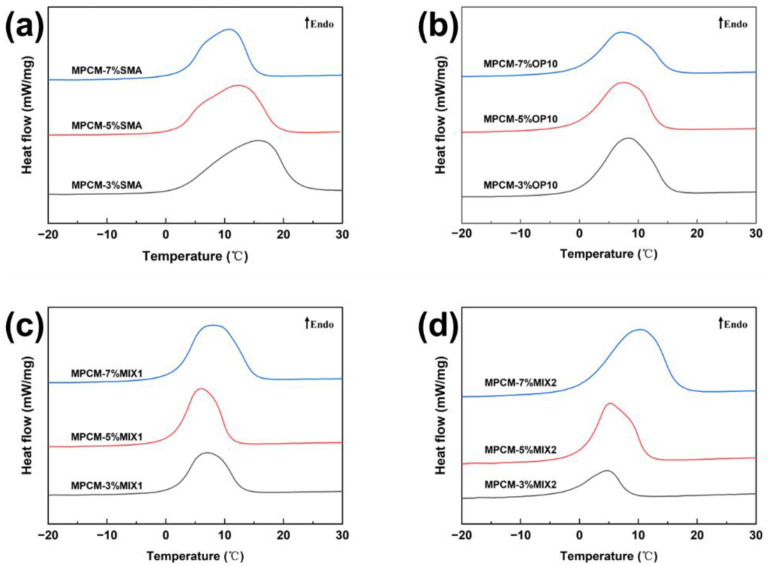
DSC curves of MPCMs prepared with different emulsifiers: (**a**) SMA, (**b**) OP10, (**c**) 15 wt% Span80/85 wt% Tween80, (**d**) 75 wt% Span80/25 wt% SDS.

Maintaining the stability of the emulsion system is essential for successful polymerization. In an emulsion polymerization system, the addition of an emulsifier reduces interfacial energy, allowing the generated emulsion particles to be stably dispersed in the medium and form a polymer emulsion in a thermodynamically sub-stable state. 1-decanol was used as the core material while keeping other variables constant, varying only the types and dosages of emulsifiers. Figure 2 shows the DSC curves of microcapsules prepared with different emulsifiers and dosages, while Table 2 presents the specific thermophysical properties of each group of MPCMs. Among the four emulsifiers tested, MPCMs stabilized with SMA exhibited the highest enthalpy, with the group containing 3% SMA in the oil phase showing the highest encapsulation rate of 56.8% among all experimental groups.

The thermal properties of MPCMs and the dosage of the compound emulsifier showed a positive correlation trend. At low emulsifier concentrations, only a portion of the latex particle surface is covered by emulsifier molecules. Under such conditions, latex particles tend to aggregate or adhere, leading to poor dispersion of the MPCMs. Additionally, the shell material cannot effectively form a core–shell structure, resulting in low phase-change enthalpy of MPCMs. As the emulsifier concentration increases, the oil–water interfacial tension is reduced, and emulsifier molecules rapidly diffuse to newly formed interfaces. This rapid coverage by a monomolecular layer of surfactant stabilizes the interface and prevents droplet recombination. A stronger stabilization tendency leads to better emulsifier performance, generating more stable emulsion systems with smaller particle sizes and better dispersion of microcapsules after polymerization. The phase-change enthalpy of MPCMs decreased with higher SMA and OP10 doping. Compared to larger MPCMs of the same mass, smaller MPCMs contain less core material, leading to lower phase-change enthalpy. Additionally, the suspension polymerization method used in this study requires the emulsifier dosage to remain below the critical micelle concentration (CMC). As a result, solid particles are formed, leading to a reduced encapsulation rate of the microcapsules [11,17].

**Figure 3:**
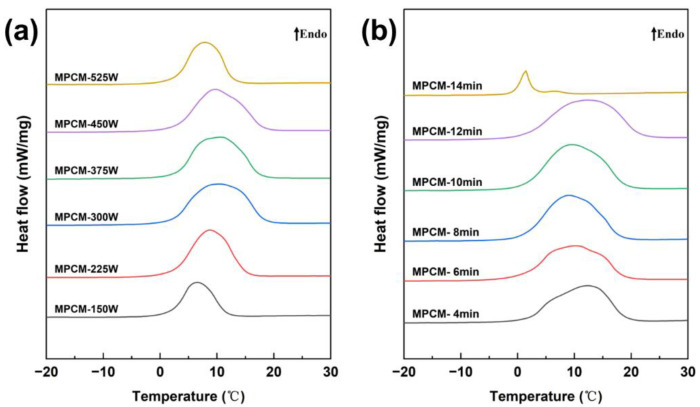
DSC curves of MPCMs prepared with different ultrasonic dispersion parameters: (**a**) ultrasound power, (**b**) ultrasound time.

The ultrasonic dispersion time was set to 8 min to study the effect of power, while the power was fixed at 375 W to investigate the effect of time. Figure 3 shows the DSC curves of MPCMs prepared under different ultrasonic dispersion parameters, and the specific thermophysical properties are summarized in Table 3. As ultrasonic power increased, the emulsification efficiency improved, resulting in a more homogeneous emulsion and higher phase-change enthalpy of MPCMs. The encapsulation rate reached 56.1% when the power was 300 W. Further increases in power to 375 W and 450 W caused no significant change in the encapsulation rate. However, at 525 W, the encapsulation rate decreased significantly. This decline was due to excessive heat generation, which raised the system temperature, destabilized the emulsion, and reduced the enthalpy of the MPCMs. At lower ultrasonic power, the system failed to form a uniform O/W emulsion, resulting in poor emulsification and low phase-change enthalpy. Increasing the ultrasonic power improved emulsification up to a point, but excessive power had adverse effects. As ultrasonic dispersion time increased, the emulsion became more homogeneous, and the encapsulation rate gradually improved, exceeding 60% at 10 and 12 min. However, at 14 min, the high temperature caused by prolonged sonication triggered burst polymerization, producing a large number of small particles. As a result, the encapsulation rate dropped sharply to 6.5%. This was because the burst polymerization consumed the monomers prematurely, leaving no surplus monomers available to cover the core material during the polymerization stage.

### 3.2. Effects of Emulsification Process on Micromorphology

**Figure 4:**
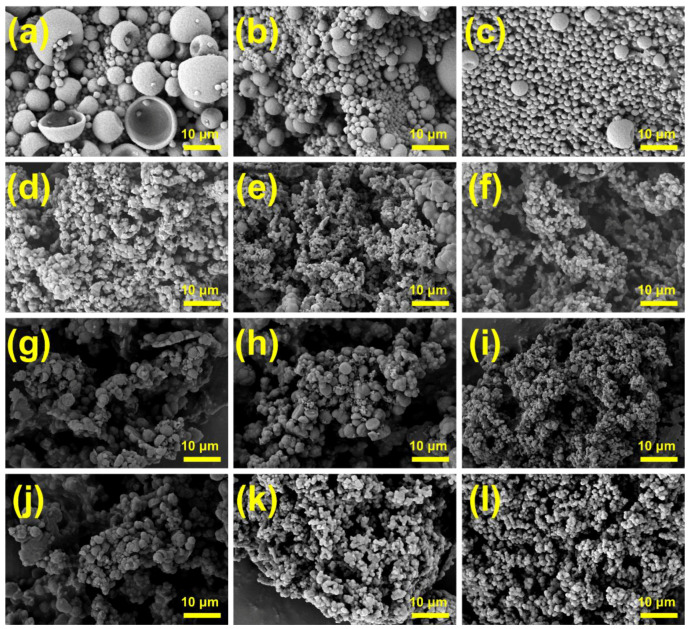
SEM images of MPCMs prepared with different emulsifiers: (**a**–**c**) 3%, 5%, and 7% SMA, (**d**–**f**) 3%, 5%, and 7% OP10, (**g**–**i**) 3%, 5%, and 7% MIX1, (**j**–**l**) 3%, 5%, and 7% MIX2.

Figure 4 presents microscopic images of MPCMs prepared using different types and dosages of emulsifiers. Among the four emulsifier groups, MPCMs prepared with SMA exhibited the optimal morphology. The remaining three groups showed irregular particle shapes or significant particle aggregation. SMA functions not only as an emulsifier but also as a protective colloid. Protective colloid molecules adsorb onto the emulsion particle surfaces, forming a hydration layer that prevents particle collision and premature polymerization. Additionally, SMA dissolved in the aqueous phase increases system viscosity, further reducing collisions between latex particles. As an anionic emulsifier, SMA generates hydration effects, causing electrostatic repulsion among latex particles. The combined steric and electrostatic stabilization effects result in stable emulsions and yield MPCMs with spherical and regular shapes (Figure 4a–c), consistent with the optimal DSC results shown in Figure 2 [24,25]. In the comparison of Figure 4d–f, microcapsules prepared using 3% OP10 exhibited more severe aggregation than those prepared using 7% OP10. However, the data in Table 2 indicate a higher phase-change enthalpy for microcapsules with 3% OP10. This apparent contradiction is explained by the poor encapsulation of core materials by MMA. Instead, the severe particle aggregation forms a porous skeleton structure, which temporarily enhances core-material adsorption and improves the initial thermal storage performance in DSC tests. Nevertheless, after repeated DSC cycles, the enthalpy significantly reduces. Conversely, for samples prepared with 7% OP10, the MPCM particle size became very small, which led to a lower encapsulation efficiency (as described in Section 3.1) as fewer core materials were encapsulated for the same sample mass. The significant aggregation observed for MPCMs prepared with mixed emulsifiers can be attributed to incomplete mixing of emulsifier components before preparation. Moreover, the composite emulsifiers likely exhibited an unsuitable HLB value relative to that required for stable O/W emulsions, thus failing to achieve effective stabilization during the emulsification process.

**Figure 5:**
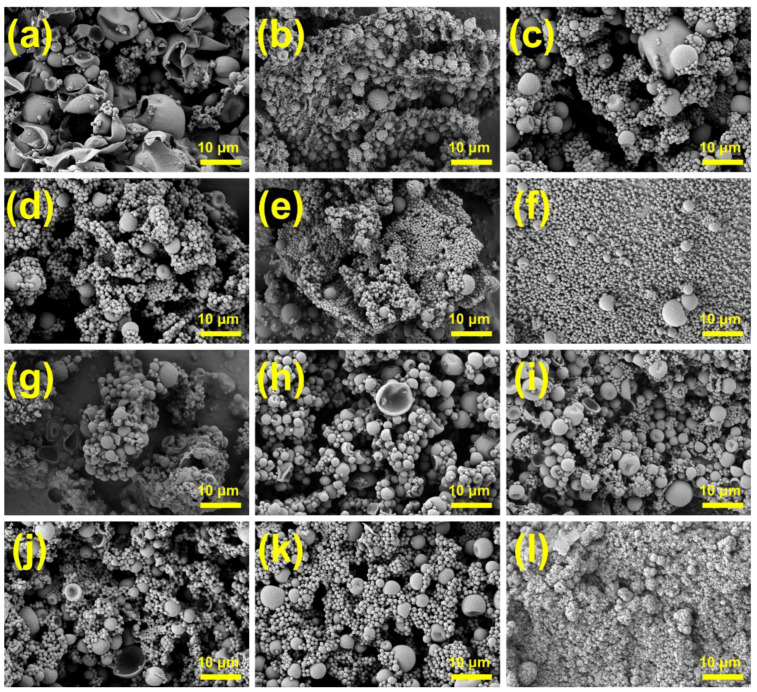
SEM images of MPCMs prepared with different ultrasonic dispersion parameters: (**a**) 150 W, (**b**) 225 W, (**c**) 300 W, (**d**) 375 W, (**e**) 450 W, (**f**) 525 W, (**g**) 4 min, (**h**) 6 min, (**i**) 8 min, (**j**) 10 min, (**k**) 450 W, (**l**) 12 min.

As shown in Figure 5a, larger MPCMs exhibit significant damage, depressions, and wrinkles due to thermal expansion and contraction, corresponding to the lower phase-change enthalpy values shown in Table 3. Increasing the ultrasound duration produces a more stable and uniform emulsion, significantly enhancing the morphology of microcapsules (Figure 5b). With increasing ultrasonic power, MPCMs come to be smaller and better dispersed. However, Table 3 shows that the encapsulation efficiency begins to decrease when the ultrasonic power exceeds 300 W. Specifically, at 525 W, the encapsulation efficiency decreases by approximately 15% compared to that at 300 W. As can be seen by comparing Figure 5d with Figure 5f, the reduced particle size increases the specific surface area, thus enhancing thermal conductivity and shortening the phase-transition interval. However, such smaller capsules encapsulate less core material, resulting in a lower encapsulation efficiency. The poor dispersion and irregular particle shapes in Figure 5g are attributed to a short emulsification time and instability of the emulsion system. In contrast, Figure 5h–k show numerous large-sized microcapsules, possibly due to Ostwald ripening. During this phenomenon, smaller droplets with higher surface energy diffuse and merge into larger ones, giving rise to large microcapsules during polymerization.

In Figure 5l, no clear core–shell microcapsule structure can be observed. This is due to prolonged ultrasonication, causing an excessive system temperature and thus triggering burst polymerization. This explains the minimal DSC peak and very low phase-change enthalpy which can be seen for MPCMs prepared with a 14 min ultrasonication time in Figure 3b.

### 3.3. Composition and Structure of MPCMs

**Figure 6:**
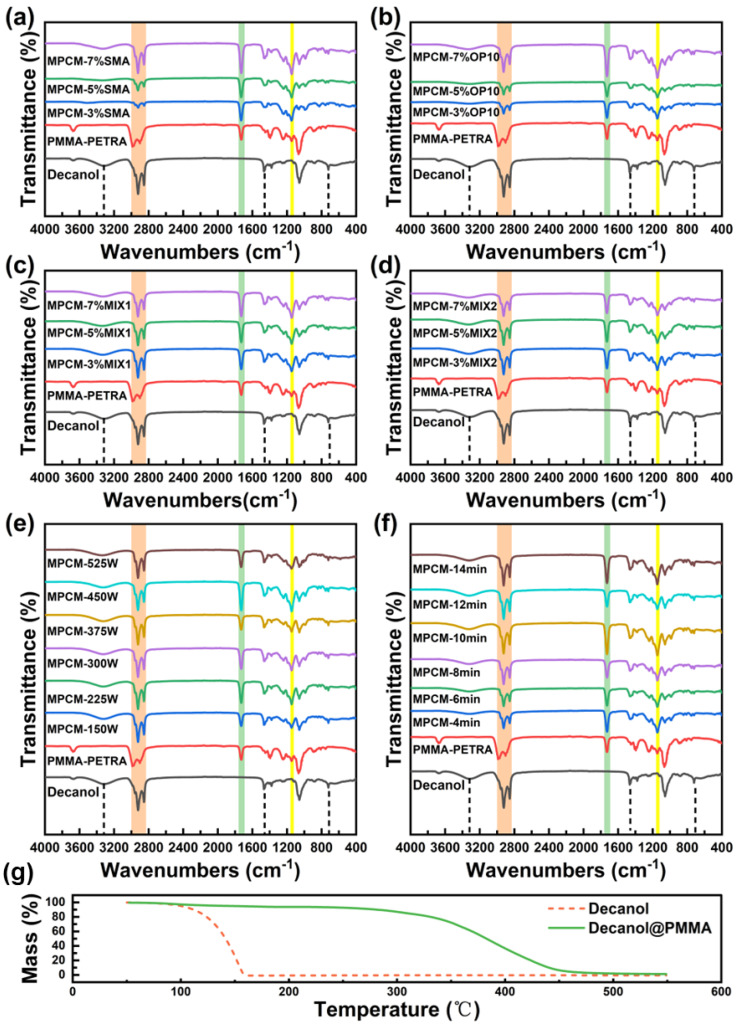
(**a**–**f**) Infrared spectra of MPCMs prepared using different ultrasonic dispersion emulsification processes, (**g**) thermogravimetric curves of 1-decanol@PMMA and pure 1-decanol.

Figure 6a–f show the infrared spectra of different MPCMs. Compared with pure 1-decanol, 1-decanol@PMMA exhibits distinct characteristic peaks. It can be seen that the O-H stretching vibration peak at 3325 cm^−1^ is of 1-decanol, the bending vibration peak of O-H at 720 cm^−1^ is of 1-decanol, the 2925 cm^−1^–2850 cm^−1^ multiple absorption peaks are the stretching vibration peaks of methyls and methylene in the aliphatic group, and there is also a bending vibration peak of C-H at 1464 cm^−1^. Since the microcapsule shells consist of PMMA–PETRA crosslinked copolymer, the 1-decanol microcapsules present new characteristic peaks not observed in pure 1-decanol; these include the C=O stretching vibration peak at 1730 cm^−1^ associated with PMMA, and the characteristic C-O-C stretching and bending vibrations from PETRA at 1148 cm^−1^ [20]. Peaks at 2926 cm^−1^ and 2853 cm^−1^ correspond to the stretching vibrations of methyl and methylene groups in the polymer shell [5,23]. Thus, according to the infrared results and SEM morphology (Figure 4 and Figure 5), the microcapsule shells are confirmed as PMMA–PETRA crosslinked copolymers. Furthermore, no chemical reaction took place between the 1-decanol core and the polymer shell, demonstrating that 1-decanol remains encapsulated as a stable core material within the polymeric microcapsule.

Figure 6g shows the thermogravimetric curves of 1-decanol@PMMA. Pure 1-decanol exhibited almost complete mass loss (nearly 100%) at 158.8 °C, while the microcapsule shell began to decompose at 332.6 °C. These results indicate that the core–shell structure effectively prevents leakage of the PCMs and improves its thermal stability; this result is consistent with previous studies [25,45]. This highlights the advantages of the microencapsulation process and expands its application range.

**Figure 7:**
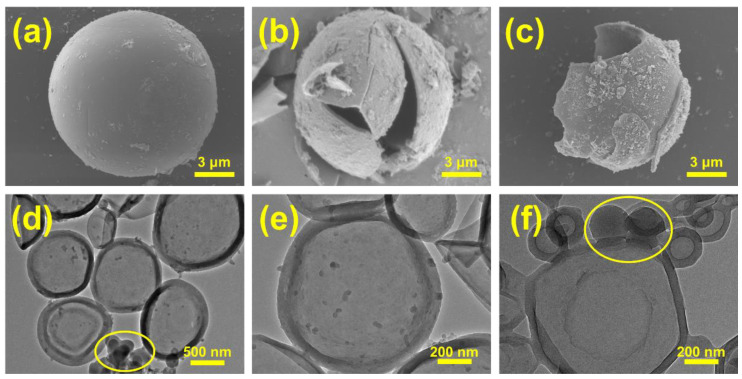
Microstructural diagram of 1-decanol@PMMA: (**a**–**c**) SEM images, (**d**–**f**) TEM images.

**Figure 8:**
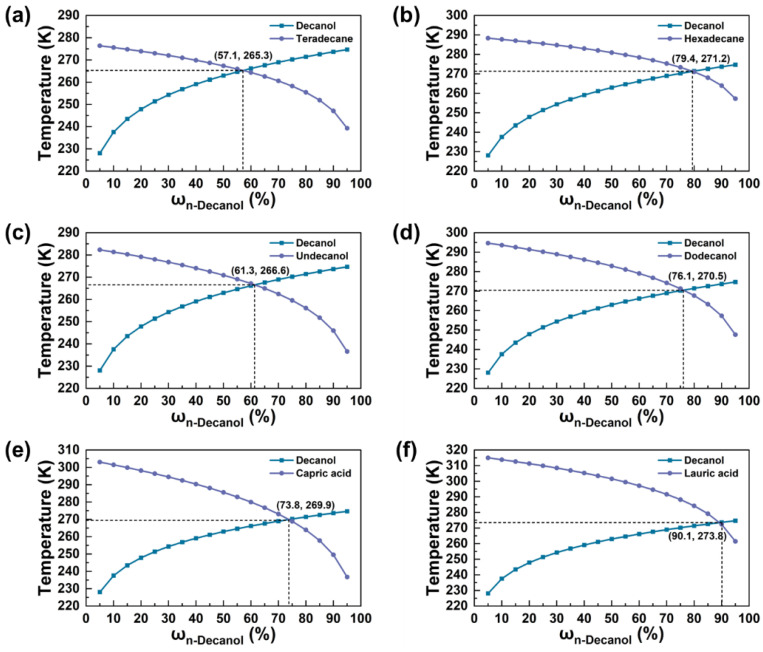
Phase diagram of binary eutectic PCMs: (**a**) 1-decanol/1-tetradecane, (**b**) 1-decanol/1-hexadecane, (**c**) 1-decanol/1-undecanol, (**d**) 1-decanol/1-dodecanol, (**e**) 1-decanol/capric acid, (**f**) 1-decanol/lauric acid.

Figure 7a–c show SEM images of individual microcapsules. A clear core–shell structure can be observed from the broken shells, where a polymer shell composed of PMMA encapsulates the PCM. Figure 7d–f present TEM images of 1-decanol@PMMA, further confirming the core–shell structure and good dispersibility [46]; 1-decanol@PMMA observed by TEM appeared less spherical than in SEM images, due to deformation under the high-energy electron beam at greater magnifications. In Figure 7d,f (marked circles), the small-sized microcapsules exhibit a darker contrast than the larger ones, indicating less core material was encapsulated inside. Figure 8f shows that the shell material of small-sized microcapsules occupies a relatively large volume fraction in the MPCMs. Additionally, the high concentration of SMA and OP10 used as emulsifiers leads to the formation of numerous smaller solid particles, which subsequently reduces the overall phase-change enthalpy of MPCMs shown in Figure 2.

### 3.4. Enhancement of Thermal Properties of Microcapsules by Binary Eutectic Method

In this study, 1-tetradecane, 1-hexadecane, 1-undecanol, 1-dodecanol, capric acid, and lauric acid are separately combined with 1-decanol to form binary eutectics. The eutectic points of binary eutectic cores were determined by plotting binary phase diagrams. The thermal properties of the eutectic points are shown in Table 4. As shown in Figure 8, these eutectic points after compounding are all below 0 °C. Therefore, the peak temperatures of the binary eutectic cores lie within the temperature range of 2–8 °C. This facilitates heat absorption by the MPCMs within the 2–8 °C interval, thus enhancing the cold-storage performance of the materials.

**Figure 9:**
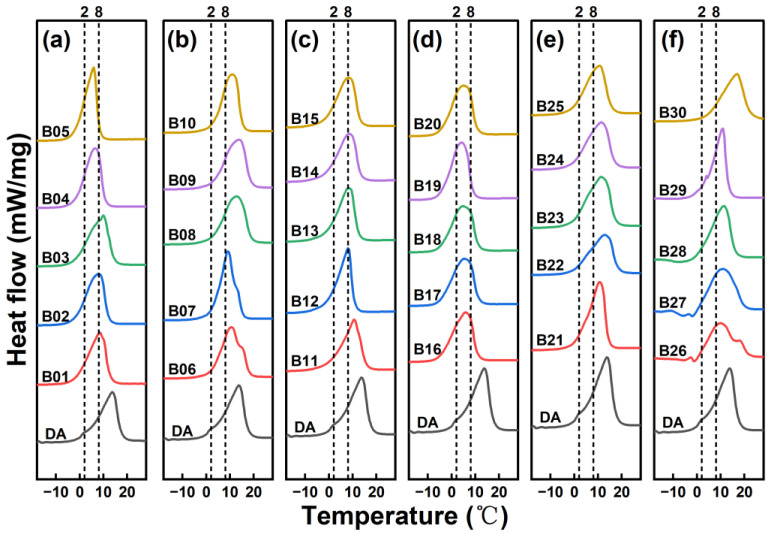
DSC curves of binary eutectic PCMs: (**a**) 1-decanol/1-tetradecane, (**b**) 1-decanol/1-hexadecane, (**c**) 1-decanol/1-undecanol, (**d**) 1-decanol/1-dodecanol, (**e**) 1-decanol/capric acid, (**f**) 1-decanol/lauric acid.

The resulting DSC curves are shown in Figure 9. The related thermophysical properties are summarized in Table 5. The eutectic mixtures of 1-decanol/1-tetradecane and 1-decanol/1-dodecanol exhibited a suitable phase-change performance for cold storage. Specifically, the target temperature interval of 2–8 °C was located within the phase-transition region of these PCMs. According to the phase-transition enthalpy values provided in Table 5, the eutectic mixture of 1-decanol and 1-tetradecane showed a relatively higher phase-change enthalpy. The phase-change temperature ranges of 1-hexadecane mixtures did not exhibit the theoretically expected eutectic points. Moreover, the DSC curve for the lauric acid and 1-decanol combination showed multiple minor peaks, indicating that substances with significantly different phase-transition temperatures do not demonstrate good compatibility when blended.

### 3.5. Properties of MPCMs Prepared by Binary Eutectic Cores

Based on the results obtained in Section 3.1, Section 3.2, and Section 3.4, high-performance cold-storage phase-change microcapsules were prepared. SMA doped with 5% emulsifier was used, ultrasonic power was set to 375 W, and ultrasonic treatment lasted for 10 min. A binary eutectic mixture of 1-decanol and 1-tetradecane was selected as the core material.

**Figure 10:**
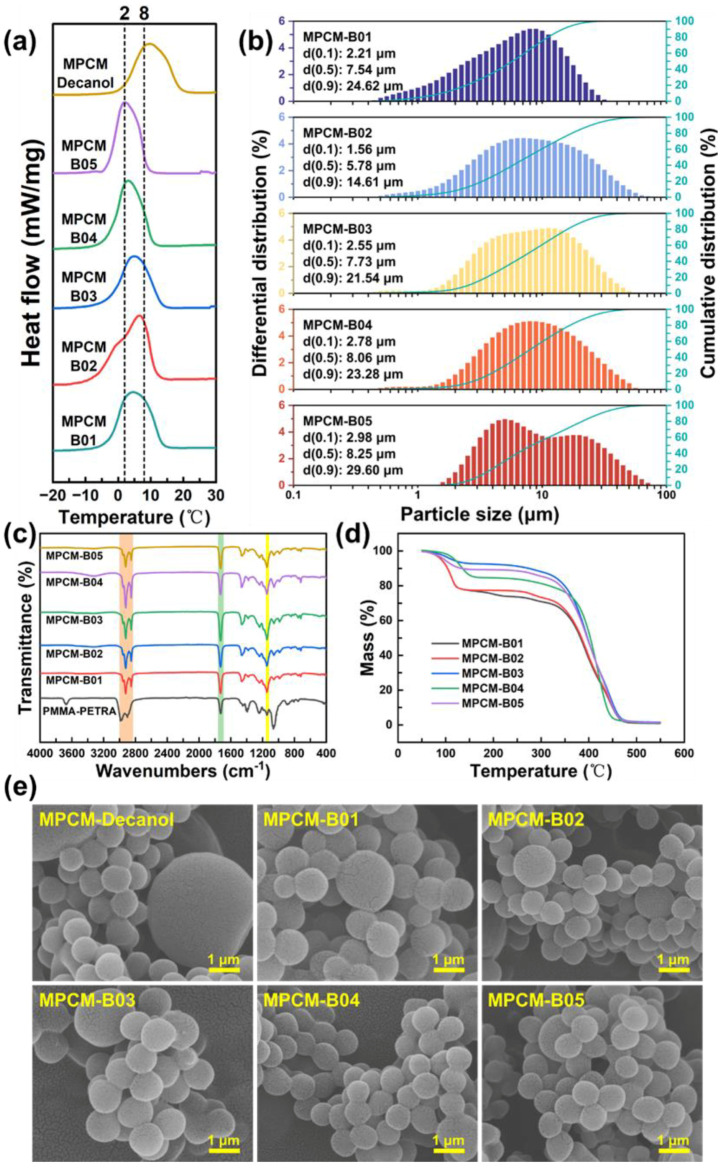
Properties of binary-core MPCMs: (**a**) DSC curves, (**b**) particle size distribution, (**c**) infrared spectrum, (**d**) thermogravimetric curves, (**e**) SEM images.

As shown in Table 6 and Figure 10a, the binary-core MPCMs exhibit an improved thermal performance. In addition to a phase-change temperature better matching the required cold-storage temperature range, binary-core MPCMs also have a higher phase-change enthalpy [37]. The phase-change enthalpy of binary-core MPCMs in group B01 (144.3 kJ/kg) is 13.9% higher than that of the 1-decanol MPCMs with the maximum enthalpy value (126.7 kJ/kg) listed in Table 3. This enhancement may be attributed to the superior thermal properties of 1-tetradecane compared to 1-decanol.

Figure 10b shows the particle size distribution of the binary-core microcapsules, and the d(0.5) of all the samples is less than 10 μm. However, as indicated by Figure 4, Figure 5 and Figure 10e, the particle sizes observed via SEM are smaller than those measured by laser particle sizing. This discrepancy may be caused by the aggregation of MPCMs, or possibly insufficient dispersion time during sample preparation. Both factors can lead to agglomeration of MPCMs, thus resulting in larger measured particle sizes.

The infrared spectrum in Figure 10c shows the characteristic stretching vibration peak of C=O from PMMA at 1730 cm^−1^. The stretching and bending vibration peaks of the C–O–C group from PETRA appear at 1148 cm^−1^. Additionally, the stretching vibration peaks of substituent and methylene groups occur at 2926 cm^−1^ and 2853 cm^−1^, respectively. These results, combined with the SEM image in Figure 10e, indicate that the polymer shell effectively encapsulates the core PCMs.

Figure 10d shows the thermogravimetric curves of the binary-core MPCMs. Compared with the core PCMs shown in Figure 6g, MPCMs exhibit significantly improved thermal stability. This improvement indicates an effective protective role of the polymer shell. At higher temperatures, MPCMs initially undergo thermal decomposition of the core material, leading to partial rupture of the shell. As temperature increases further, the polymer shell begins decomposing at approximately 300–400 °C and completely decomposes at between 500 and 600 °C.

Figure 10e presents the SEM images of MPCMs. Under optimal preparation conditions, MPCMs exhibit good dispersion, a regular morphology, and a relatively uniform spherical shape. Combined with Figure 5 and Figure 6, it can be seen that MPCMs prepared with 1-decanol as the core material contain more large-sized particles. This effect likely results from Ostwald ripening during emulsification. Because alcohol can dissolve emulsifiers, their presence in the emulsion can reduce emulsion stability. Emulsifiers migrate from the droplet surface into the aqueous phase, accelerating Ostwald ripening. Consequently, as mentioned in Section 3.2, smaller droplets with higher surface energy merge into larger droplets. This phenomenon results in large-sized microcapsules during polymerization. SEM images reveal a few large microcapsules mixed with many smaller capsules. However, this issue is significantly improved by using binary eutectic PCMs as the core material, where 1-tetradecane acts as a stabilizing component [47].

## 4. Conclusions

In this study, MPCMs were synthesized by suspension polymerization with an optimized emulsification process, which simplified the preparation procedure. The prepared MPCMs showed high phase-change enthalpy, good dispersion, and a smooth spherical morphology. A binary eutectic method was used to adjust the core PCM and obtain the desired phase-transition temperature range for low-temperature refrigeration at 2–8 °C, thus better meeting cold-chain temperature requirements. This method also significantly improved the phase-change enthalpy. A binary eutectic mixture of 1-decanol and 1-tetradecane (molar ratio 51.1:48.9) was selected as the core material. Optimal emulsification was achieved with an SMA emulsifier dosage of 5%, ultrasonic power of 375 W, and ultrasonic time of 10 min. Under these conditions, the MPCMs exhibited a high phase-change enthalpy of 144.3 kJ/kg, indicating a superior cold-storage performance in low-temperature applications. Scanning electron microscopy indicated that these MPCMs possessed a regular spherical shape and good encapsulation quality, leading to a longer cycle life and enhanced practical applicability. Overall, these binary-eutectic-core MPCMs demonstrate good thermal stability, high enthalpy, and stable cycling characteristics, suggesting their strong potential for cold-chain transportation, packaging, and other cold-storage uses.

## Figures and Tables

**Table 1:** Emulsifiers used in the experiment and their HLB values.

Samples	Emulsifiers	Ionic Properties	HLB
SMA	100 wt% SMA	Anionic	15.4
OP10	100 wt%OP10	Nonionic	13.3~14.0
MIX1	15 wt% Span80/85 wt% Tween80	Nonionic/Nonionic	13.4
MIX2	75 wt% Span80/25 wt% SDS	Nonionic/Anionic	13.2

**Table 2:** Thermophysical properties of MPCMs using different types and dosages of emulsifiers.

Samples	Onset (°C)	Peak (°C)	Endset (°C)	ΔH_m_ (kJ/kg)	E (%)
MPCM-3%SMA	1.8	15.5	22.0	118.4	56.8
MPCM-5%SMA	2.3	12.9	19.5	95.1	45.6
MPCM-7%SMA	2.8	10.6	15.3	74.8	35.9
MPCM-3%OP10	0.4	8.3	15.5	101.9	48.8
MPCM-5%OP10	−0.2	7.2	13.6	92.8	44.5
MPCM-7%OP10	−0.1	7.0	15.7	86.5	41.5
MPCM-3%MIX1	1.7	7.1	12.9	58.9	28.2
MPCM-5%MIX1	1.2	6.0	11.0	71.0	34.0
MPCM-7%MIX1	1.6	7.8	15.2	95.8	45.9
MPCM-3%MIX2	−2.4	4.6	8.5	26.1	12.5
MPCM-5%MIX2	0.5	5.1	11.5	60.8	29.1
MPCM-7%MIX2	0.3	10.3	16.7	98.7	47.3

**Table 3:** Thermophysical properties of MPCMs using different ultrasonic dispersion parameters.

Samples	Onset (°C)	Peak (°C)	Endset (°C)	ΔH_m_ (kJ/kg)	E (%)
MPCM-150W	2	6.5	11.6	53.4	25.7
MPCM-225W	2.5	8.7	14.9	103.6	49.8
MPCM-300W	2.1	10.3	18.1	116.6	56.1
MPCM-375W	3	10.6	17.6	113.9	54.8
MPCM-450W	2.9	9.7	18.2	110.1	52.9
MPCM-525W	2.9	7.8	12.6	81.8	39.3
MPCM-4 min	1.8	12.4	19	95.1	45.7
MPCM-6 min	1.2	10.2	18.5	104.4	50.2
MPCM-8 min	1.2	9	18.3	119.4	57.4
MPCM-10 min	1.4	9.7	19	126.1	60.6
MPCM-12 min	1.4	12.2	21.5	126.7	60.9
MPCM-14 min	−0.5	1.4	2.7	13.6	6.5

**Table 4:** The theoretical eutectic points of binary eutectic mixtures formed separately by 1-decanol with various different phase-change materials.

Component 1	Mole Fraction (%)	Component 2	Mole Fraction (%)	Eutectic Point (K)
1-decanol	57.1	1-tetradecane	42.9	265.3
1-decanol	79.4	1-hexadecane	20.6	271.2
1-decanol	61.3	1-undecanol	38.7	266.6
1-decanol	76.1	1-dodecanol	23.9	270.5
1-decanol	73.8	Capric acid	26.2	269.9
1-decanol	90.1	Lauric acid	9.9	273.8

**Table 5:** Actual thermophysical data of binary eutectic mixtures formed separately by 1-decanol with various different phase-change materials.

Samples	Components	Mole Ratio	Onset (°C)	Peak (°C)	Endset (°C)	ΔH_m_ (kJ/kg)
B01	Decanol/Tetradecane	51.1:48.9	−1.8	8.5	12.4	215.9
B02		54.1:45.9	−2.1	8.0	12.0	221.0
B03		57.1:42.9	−2.2	10.0	14.5	231.6
B04		60.1:39.9	−1.9	6.5	10.5	225.0
B05		63.1:36.9	−2.2	5.9	8.2	217.3
B06	Decanol/Hexadecane	73.4:26.6	2.0	10.6	18.7	214.8
B07		76.4:23.6	2.6	9.3	13.3	223.7
B08		79.4:20.6	2.6	12.8	18.8	216.8
B09		82.4:17.6	2.9	13.8	18.7	221.2
B10		85.4:14.6	3.2	10.8	15.1	209.8
B11	Decanol/Undecanol	55.3:44.7	0.8	10.8	15.8	194.0
B12		58.3:41.7	−0.7	8.1	10.6	192.7
B13		61.3:38.7	−1.1	8.1	12.4	195.3
B14		64.3:35.7	−1.7	8.6	13.2	190.4
B15		67.3:32.7	−2.0	8.3	13.0	196.3
B16	Decanol/Dodecanol	70.1:29.9	−5.0	5.8	10.8	180.5
B17		73.1:26.9	−5.0	5.5	11.1	180.6
B18		76.1:23.9	−4.7	4.8	11.0	176.8
B19		79.1:20.9	−4.4	4.3	8.8	183.4
B20		82.1:17.9	−4.3	5.1	10.4	183.5
B21	Decanol/Capric acid	67.8:32.2	1.8	10.8	14.2	186.3
B22		70.8:29.2	−1.1	12.8	18.2	150.0
B23		73.8:26.2	−1.1	11.4	17.0	189.7
B24		76.8:23.2	−1.0	11.4	16.7	174.2
B25		79.8:20.2	−0.9	10.6	15.6	169.6
B26	Decanol/Lauric acid	84.1:15.9	0.4	22.7	22.7	149.1
B27		87.1:12.9	0.1	20.2	20.2	165.2
B28		90.1:9.9	0.3	15.9	15.9	163.2
B29		93.1:6.9	0.5	13.3	13.3	172.7
B30		96.1:3.9	3.6	22.3	22.2	180.1

**Table 6:** Thermophysical data of MPCMs using the binary eutectic core material composed of 1-decanol and 1-tetradecane.

Samples	Onset (°C)	Peak (°C)	Endset (°C)	ΔH_m_ (kJ/kg)	E (%)
MPCM-B01	−2.8	4.5	13.6	144.3	66.8
MPCM-B02	−3.7	6.5	12.8	136.8	61.9
MPCM-B03	−2.7	5.2	13.5	130.4	56.3
MPCM-B04	−2.8	3.0	10.7	135.6	60.3
MPCM-B05	−3.0	2.2	8.8	138.8	63.9

## Data Availability

The original contributions presented in this study are included in the article. Further inquiries can be directed to the corresponding authors.
